# Clinical effects of basic nursing combined with psychological intervention on treatment compliance of patients with Influenza-A(H1N1)

**DOI:** 10.12669/pjms.40.7.8675

**Published:** 2024-08

**Authors:** Yaning Feng, Chuntao Ma, Zhongxian Feng, Yajing Bian, Yujiao Zhu, Kun Li

**Affiliations:** 1 Yaning Feng, Health Management and Physical Examination Center, The First Affiliated Hospital of Hebei North University, Zhangjiakou, 075000, Hebei, China; 2 Chuntao Ma, Health Management and Physical Examination Center, The First Affiliated Hospital of Hebei North University, Zhangjiakou, 075000, Hebei, China; 3 Zhongxian Feng, Department of Nursing First School of Clinical Medicine, Hebei North University, Zhangjiakou, 075000, Hebei, China; 4 Yajing Bian, The Outpatient Department, The First Affiliated, Hospital of Hebei North University, Zhangjiakou, 075000, Hebei, China; 5 Yujiao Zhu, Health Management and Physical Examination Center, The First Affiliated Hospital of Hebei North University, Zhangjiakou, 075000, Hebei, China; 6 Kun Li, The Outpatient Department, The First Affiliated, Hospital of Hebei North University, Zhangjiakou, 075000, Hebei, China

**Keywords:** Basic nursing, Psychological intervention, Influenza-A(H1N1), Compliance, Nursing satisfaction

## Abstract

**Objective:**

To investigate the effects of basic nursing combined with psychological intervention on treatment compliance, self-care ability, clinical efficacy, lung function and nursing satisfaction of patients with Influenza-A(H1N1).

**Method:**

This was application research. Eighty patients with influenza-A (H1N1) admitted to The First Affiliated Hospital of Hebei North University from January 2020 to December 2022 were included as subjects and randomly divided into the observation group(n=40) and the control group(n=40). Patients in the control group were given routine basic nursing intervention, while those in the observation group were treated with combined psychological intervention in addition to basic nursing. The differences in treatment compliance, self-care ability, clinical efficacy, lung function and nursing satisfaction were compared between the two groups.

**Results:**

After the intervention, the treatment compliance score and the total self-care ability score of the observation group were higher than those of the control group, with statistically significant differences(P<0.05). After treatment, the clinical efficacy of the observation group was significantly higher than that of the control group(P<0.05). Before treatment, no significant difference was observed between the two groups in terms of various indexes of lung function, which were better in the observation group than in the control group after treatment(P<0.05).

**Conclusion:**

Basic nursing combined with psychological intervention results in a variety of benefits in the treatment of patients with Influenza-A(H1N1), such as improved treatment compliance and self-care ability, ameliorated lung function, as well as enhanced treatment outcomes and nursing satisfaction, which needs to be promoted in clinical practice.

## INTRODUCTION

Peripher Influenza-A (H1N1) is an acute respiratory infectious disease caused by a novel type of Influenza-A (H1N1) virus.^1^ It comprises gene fragments of three types of influenza viruses: human influenza, avian Influenza-And swine influenza, which puts the population in a vulnerable situation, and whoever is infected will suffer from a more rapid disease progression. For some patients, pneumonia, respiratory failure, and multiple organ dysfunction may occur in severe cases, endangering their lives.^2^,^3^ The disease is highly contagious and requires isolation and treatment of confirmed patients. Despite the clinical availability of a variety of preventive treatment measures for Influenza-A (H1N1), patients often have a rejection of isolation, and in severe cases, mental or emotional maladaptation and obstacles.^4^

For this reason, effective nursing and psychological treatment should be carried out while actively treating patients with influenza- (H1N1). Sound nursing methods and psychotherapy are effective medicine to improve the confidence of patients in treatment, which are used to prevent the deterioration of the disease and promote the rapid recovery of patients^5^. Basic nursing is a type of nursing that meets the needs of patients in treatment as much as possible by combining the needs of patients’ physiology, psychology and rehabilitation based on the basic theory, knowledge and skills of nursing.

It is primarily to observe the changes in the condition, provide daily nursing and guide the rehabilitation exercise of patients but ignores the intervention on the psychological changes of the patients, which affects the quality of nursing to a certain extent.^6^ If psychological intervention is carried out in addition to basic nursing, it can thoroughly mobilize patients’ subjective initiative and enthusiasm, alleviate or eliminate their negative emotions, and enhance their confidence in overcoming the disease, thereby improving clinical efficacy and nursing effects^7^. In this study, we involved basic nursing and psychological intervention together to evaluate the treatment of patients with influenza-A (H1N1), to investigate the clinical importance of applying the combined model for the treatment of the disease.

## METHODS

This was a application research. Eighty patients with Influenza-A (H1N1) admitted to The First Affiliated Hospital of Hebei North University from January 2020 to December 2022 were included as subjects and randomly divided into two groups by random number table method: the observation group and the control group, with 40 cases in each group according to different treatment methods.

### Ethical Approval

The study was approved by the Institutional Ethics Committee of The First Affiliated Hospital of Hebei North University (No.:2021071; July 16, 2021), and written informed consent was obtained from all participants.

### Inclusion criteria:

- Patients who met the diagnostic criteria of the “Influenza-A H1N1 Diagnosis and Treatment Program (2010 Edition)”^8^ and whose clinical data were complete.
- Patients with influenza-like clinical manifestations, positive throat swabs for Influenza-A (H1N1) virus nucleic acid test, and imaging findings consistent with influenza manifestations.
- Patients with good communication, informed consent of themselves and their families to the content of this study, and voluntarily signed informed consent.
- Patients with no history of allergy to therapeutic drugs.

### Exclusion criteria:

- Patients with other respiratory diseases, such as asthma.
- Pregnant or lactating women.
- Patients with critical conditions, or with serious primary diseases such as liver and kidney diseases.
- Patients with communication disorders, inability to cooperate with work, and lack of clinical data.
- Patients combined with melancholia.
- Patients with malignant tumors in other parts of the body and patients with metastatic liver cancer.

### Methods

Both groups were given standardized treatment programs such as oxygen inhalation, anti-inflammatory agents and immunostimulants. Specifically, the patients in the control group were given basic nursing, including health education for patients, enhanced communication to alleviate negative emotions, symptomatic treatment, tracheal intubation, airway nursing, aspiration of sputum, and close monitoring of vital signs as prescribed by doctors. In contrast, the patients in the observation group were given psychological intervention on top of the control group:

Patients were introduced to the hospital environment and medical staff in detail after admission to adapt to the environment faster; They were also guided to actively face the disease and establish a harmonious nurse-patient relationship after being aware of their illness and psychological conditions by the nursing staff.

Psychological intervention: Psychological assessment was carried out on the patients in the form of individual psychological intervention (2-3 times a week, 30 min each time), and individualized psychological intervention measures were formulated:

Verbal persuasion: patients were provided with psychological support and comfort, and their negative emotions are relieved.

Mental support: patients were provided with psychological support to relieve anxiety and depression as well as maintain a positive and optimistic healthy mental state.

Personalized health education: Individualized education was developed based on patients’ cognitive and educational levels, with a view to helping them establish a correct understanding of the disease and respond reasonably. During the six months follow-up of this study, the survival rate was 100%.

### Observation indexes:

***- Treatment compliance:*** It was evaluated using a self-made treatment compliance questionnaire for patients with pneumonia. The questionnaire was designed to measure patients’ compliance in three aspects: compliance with doctor’s orders, diet compliance, and daily life compliance. A total of 10 items were set, each using a 4-Point Likert Scale, with a total score of 10-40 points. A higher score indicates higher patient compliance.
***- Self-care ability:*** It was evaluated by the Exercise of Self-care Ability Scale (ESCA). The scale consists of 43 items in four dimensions, including self-concept, health knowledge level, health responsibility and self-care skills, and each item adopts a 4-Point Likert Scale, with a total score of 43-172 points. A higher score indicates a higher level of patient self-care ability. –
***- Clinical efficacy:*** Referring to the “Influenza-A H1N1 Diagnosis and Treatment Program (2010 Edition)”, the clinical efficacy is divided into cured: complete disappearance or basic relief of clinical symptoms, with a negative conversion rate of over 80% of throat swab tests; Effective: significant relief of clinical symptoms, with a negative conversion rate of 60%-80% of throat swab tests; Ineffective: no relief or worsening of clinical symptoms, with a negative conversion rate of less than 30% of throat swab tests. Overall response rate = (cured + effective) cases/total cases×100%.
***- Lung function:*** The lung function indexes of the two groups were detected before and after treatment: forced expiratory volume in the first second (FEV_1_), forced expiratory volume in the first second as a percentage of predicted value (FEV_1_%), forced vital capacity (FVC), and FEV_1_/FVC level.
***- Nursing satisfaction:*** The “Inpatient Nursing Satisfaction Scale” was used to investigate the nursing satisfaction of the two groups when the patients were discharged. The scale consists of 21 items in three dimensions, including trust relationship, professional and technical ability as well as educational relationship, and each item adopts a 5-Point Likert Scale. Each item is scored from 1-5 points from “strongly disagreed” to “strongly agreed”, and questions 2, 3, 5, 7, 10, 13, 15 and 21 are negative scoring questions. A higher score indicates higher patient satisfaction. All the questionnaire was simply handed over to the participants to fill themselves.

### Statistical analysis

All data in this study were statistically analyzed using SPSS 22.0 software. The measurement data were represented by (*χ̅*±*S*) and tested using two independent samples t-test. The sample size is estimated by 95% confidence interval. Counting data were expressed as n(%), and χ² test was employed for comparison between groups. P< 0.05 was considered to indicate a statistically significant difference.

## RESULTS

In the observation group, there were 25 males and 15 females, aged 23-55 years, with an average age of 41.78±8.34 years. In the control group, there were 23 males and 17 females, aged 25-60 years, with an average age of 43.13±10.28 years. No significant difference was observed between the two groups in the comparison of general data (P>0.05), which was comparable, Table-I.

**Table-I:** Comparison of general data between the two groups.

Item	Observation group (n=40)	Control group (n=40)	t/χ^2^	p
Age (years )	41.78±8.34	43.13±10.28	0.645	0.521
Gender (Male/Female)	25/15	23/17	0.208	0.648
Disease duration (h)	14.30±1.42	13.73±1.66	1.664	0.100
Weight (Kg)	82.68±3.39	82.85±3.22	0.230	0.819

Before the intervention, no statistically significant differences were observed in treatment compliance between the two groups (P>0.05). After the intervention, the treatment compliance in both groups was significantly improved, especially in the observation group, with a statistically significant difference (P<0.05), Table-II.

**Table-II:** Comparison of treatment compliance between the two groups (*χ̅*±*S*).

Group	n	Before treatment	After treatment
Observation group	40	28.03±3.63	36.25±2.71
Control group	40	27.63±2.71	34.43±2.95
*t*		0.559	2.883
*p*		0.578	0.005

Before the intervention, no statistically significant differences were observed in self-care ability scores between the two groups (P>0.05). After intervention, the self-concept, health knowledge level, health responsibility, self-care skills and total scores of the two groups were all higher than before intervention, and those in the observation group were higher than those in the control group, with statistically significant differences (P<0.05), Table-III. The overall response rate of the observation group was 97.50%, which was significantly higher than that of the control group (82.50%), with a statistically significant difference (P<0.05), Table-IV.

**Table-III:** Comparison of self-care ability scores before and after intervention between the two groups (*χ̅*±*S*).

Group	Self-concept		Health knowledge level		Health responsibility		Self-care skills		Total scores	

	Before intervention	After intervention	Before intervention	After intervention	Before intervention	After intervention	Before intervention	After intervention	Before intervention	After intervention
Observation group	15.70±0.52	25.88±0.97	33.85±0.77	54.73±1.58	15.68±0.57	22.50±0.93	29.68±0.57	40.40±0.98	94.90±1.43	143.50±2.38
Control group	15.55±0.75	22.45±0.99	33.60± 0.96	46.53±1.28	15.55±0.71	17.38±1.03	29.55±0.71	36.33±1.16	94.25±1.56	122.68±2.39
*t*	1.042	15.696	1.289	25.450	0.864	23.315	0.864	16.930	1.940	39.089
*p*	0.300	0.000	0.201	0.000	0.390	0.000	0.390	0.000	0.056	0.000

**Table-IV:** Comparison of clinical efficacy between the two groups [n (%)].

Group	n	Markedly effective	Effective	Ineffective	Overall response rate (%)
Observation group	40	34 (85.00)	5 (12.50)	1 (2.50)	39 (97.50)
Control group	40	29 (72.50)	4 (10.00)	7 (17.50)	33 (82.50)
*χ* ^2^					5.000
*p*					0.025

Before treatment, no statistically significant differences were observed between the two groups in terms of FEV_1_, FEV_1_%, FVC and FEV_1_/FVC (P>0.05). After treatment, FEV_1_, FEV_1_%, FVC and FEV_1_/FVC in both groups were significantly improved, and the improvement degree in the observation group was better than that in the control group, with a statistically significant difference (P<0.05), Table-V. The nursing satisfaction score in the observation group was (93.65±4.09), which was significantly higher than that in the control group (82.90±3.71), with a statistically significant difference (t=12.325, P=0.000).

**Table-V:** Comparison of lung function between the two groups (*χ̅*±*S*).

Observation indexes	Observation point	Observation group	Control group	t	p
FEV_1_ (L)	Before treatment	1.35±0.27	1.33±0.30	0.356	0.723
	After treatment*	2.18±0.25	1.70±0.24	8.637	0.000
FEV_1_%	Before treatment	41.67±3.62	42.07±4.21	0.455	0.650
	After treatment*	64.72±4.51	56.55±5.07	7.615	0.000
FVC (L)	Before treatment	2.26±0.61	2.23±0.70	0.226	0.821
	After treatment	2.87±0.62	2.52±0.50	2.724	0.008
FEV_1_/FVC	Before treatment	0.64±0.23	0.66±0.25	0.290	0.772
	After treatment*	0.79±0.17	0.69±0.13	2.943	0.004

## DISCUSSION

It was shown in this study that the treatment compliance in the observation group was significantly higher than that in the control group (P<0.05), revealing the positive impact of basic nursing combined with psychological intervention on patients at the psychological and behavioral levels. The combination of the two improved patients’ acceptance of treatment plans and ameliorated behavioral habits, thus improving their treatment compliance. Compared with before treatment, the self-concept, health knowledge level, health responsibility, self-care skills and total scores in both groups were higher than before the intervention, and those in the observation group were higher than those in the control group (P<0.05), revealing the benefits of basic nursing combined with psychological intervention in improving patients’ cognition of disease, confidence in overcoming disease, and self-care ability. The clinical efficacy, lung function improvement and nursing satisfaction in the observation group were all higher than those in the control group (P<0.05), revealing the satisfactory intervention effect of basic nursing combined with psychological intervention on patients with Influenza-A (H1N1) and its benefits of improving treatment outcomes and nursing satisfaction.

Influenza-A (H1N1), as a respiratory disease with a high infection rate and transmission, is mainly transmitted by droplets, and is characterized by acute onset and strong infectivity. If not controlled in time, the disease progresses rapidly, which is easy to cause serious complications such as severe pneumonia.^9^-^11^ If not controlled in time, the disease will progress quickly and will easily lead to severe complications such as severe pneumonia. If it is systematically treated, patients can generally be cured clinically by drug treatment. During the treatment process, patients are required to strictly follow the doctor’s treatment regimens, such as isolation treatment. However, quite a few patients often show poor treatment compliance due to various reasons, such as drug side effects, disease symptoms and unbearable isolation.^12^,^13^

Traditional basic nursing explains H1N1-related knowledge to patients but often ignores adverse psychological problems in the treatment process. As a result, patients suffer from low treatment compliance and impaired clinical efficacy, leading to tension in the doctor-patient relationship. If supplemented with psychological intervention, which is patient-centered and focuses on the psychological state and needs of patients by means of psychological counseling, psychotherapy and cognitive behavioral therapy, it provides momentum for patients to adjust their psychological state, reduce anxiety and depression, and enhance their psychological adaptability.^14^ Studies^15^,^16^ have proved the dual role of psychological intervention in highlighting patients’ psychological state and improving their quality of life. Basic nursing and psychological interventions complement each other, the former provides patients with the necessary medical conditions, while the latter improves their ability to cope with the disease and quality of life. Together, the two contribute to the overall physical and psychological care of patients, forming comprehensive medical nursing.^17^

By analyzing the reasons for the above-mentioned benefits brought by the combination of basic nursing and psychological intervention, we believe that on the one hand, basic nursing puts a high premium on individual care and health education of patients, and guides them to use drugs correctly and take personal protective measures via detailed instructions and demonstrations, thereby improving their compliance with treatment. On the other hand, psychological intervention can help patients establish a positive treatment attitude and confidence via individual psychological education and psychological support, so as to alleviate their fear and anxiety.^18^ In comparison, basic nursing combined with psychological intervention can not only provide patients with basic living needs and a comfortable medical environment^19^, but also take into account their psychological state, prompting them to better realize and accept treatment regimens, and enhance their treatment compliance. Psychological intervention plays a vital role in effectively alleviating patients’ negative emotions and improving nursing and clinical outcomes. In this process, medical staff not only attach importance to patients’ disease recovery and psychological state but also think highly of patients’ self-efficacy improvement and self-protection.^20^ They spare no effort to explain to patients the knowledge about Influenza-A (H1N1) and the problems that may arise in the treatment process, reducing patients’ uncertainty about disease treatment and boosting their confidence in overcoming H1N1.

### Limitations

It includes a small number of observed cases and a short follow-up period. To address this, more samples should be included and follow-up time should be prolonged in follow-up studies to further analyze the impact of basic nursing combined with psychological intervention on the treatment of Influenza-A (H1N1).

## CONCLUSIONS

Basic nursing combined with psychological intervention produces positive changes in patients’ mental state and behavior habits, provides strong support for patients’ rehabilitation, and intervenes well for patients with H1N1. The combination of the two results in a variety of benefits in the treatment of Influenza-A (H1N1), such as ameliorated patient psychological status, enhanced treatment compliance and self-care ability, and improved clinical treatment outcomes and nursing satisfaction. Basic care combined with psychological intervention is undoubtedly an effective strategy for the treatment of Influenza-A (H1N1).

### Authors’ Contributions:

**YF** and **CM:** Carried out the studies, data collection, drafted the manuscript, are responsible and accountable for the accuracy and integrity of the work.

**ZF** and **YB:** Performed the statistical analysis and participated in its design.

**YZ** and **KL:** Participated in acquisition, analysis, or interpretation of data and drafting the manuscript.

All authors read and approved the final manuscript.
